# Functional and Nutritional Properties of Lion’s Mane Mushrooms in Oat-Based Desserts for Dysphagia and Healthy Ageing

**DOI:** 10.3390/foods14234153

**Published:** 2025-12-03

**Authors:** Samiddhi Gunathilake, Supuni Aluthge, Asgar Farahnaky, Tien Huynh, Geoffrey Ssepuuya, Mahsa Majzoobi

**Affiliations:** 1 Department of Food Technology and Nutrition, School of Science, STEM College, RMIT University, Bundoora West Campus, Plenty Road, Melbourne, VIC 3083, Australia; 2 Department of Biology, School of Science, STEM College, RMIT University, Bundoora West Campus, Plenty Road, Melbourne, VIC 3083, Australia; 3 Department of Food Science and Technology, Faculty of Science, Kyambogo University, Kyambogo, Kampala P.O. Box 1, Uganda

**Keywords:** Lion’s Mane, functional food, oat milk dessert, food fortification, dysphagia

## Abstract

*Hericium erinaceus* (Lion’s Mane mushroom) is a medicinal species recognised for its neuroprotective and antioxidant properties. This study investigated its potential as a functional ingredient in oat milk-based desserts formulated for individuals with dysphagia. Freeze-dried Lion’s Mane powder (LMP), containing high-quality protein (~16%, amino acid score 88%), dietary fibre (~31%), and phenolic compounds (72.15 mg GAE/g), was incorporated at varying levels using gelatin or iota-carrageenan (IC) as gelling agents. Incorporation of up to 5% LMP significantly improved the nutritional composition and maintained favourable texture and sensory characteristics while meeting the International Dysphagia Diet Standardisation Initiative (IDDSI) Level 6 criteria. Both manual and instrumental fork pressure tests confirmed that all samples were soft and easy to compress without stickiness or deformation recovery, ensuring safe swallowing. Higher inclusion levels of LMP or hydrocolloids increased hardness and firmness but adversely affected colour and mouthfeel. Carrageenan-based formulations further supported the development of vegan-friendly options with stable structure and desirable rheology. Overall, the study demonstrates that Lion’s Mane-enriched soft foods can deliver enhanced nutrition and texture suitable for dysphagic diets, offering a novel, plant–fungal approach to supporting healthy ageing with potential neuroprotective properties.

## 1. Introduction

*Hericium erinaceus* (Lion’s Mane) is an edible mushroom also known by various names such as Yamabushitake, Monkey’s Mushroom, Old Man’s Beard, Bearded Tooth, Houtou, and Pom Pom. It belongs to the class Basidiomycetes, order Hericiales, and family *Hericiaceae* [1].It is widely consumed in East Asia, particularly in Japan and China, due to its nutritional and medicinal properties. Traditionally used in Chinese medicine, it is recognised for its potential to prevent and treat oxidative stress-related disorders [2,3] (Ghosh et al., 2021; Szydłowska-Tutaj et al., 2023). *H. erinaceus* is a saprotrophic or a weak parasitic fungus, typically found on decaying hardwood or within tree cavities. Its mature fruiting body is fleshy, semi-spherical, and white, with long, hanging spines that may yellow or brown with age [1,4].

*H. erinaceus* exhibits a wide range of medicinal properties, including antimicrobial, anticarcinogenic, antidiabetic, antihypertensive, antihyperlipidemic, neuroprotective, and cognitive-enhancing effects [2,5]. These therapeutic benefits are attributed to bioactive compounds such as polysaccharides, β-glucans, lectins, phenolic acids, and terpenoids (hericenones and erinacines) found in both its fruiting bodies and mycelia [3,4]. These compounds support neurogenesis, immune modulation, and circulatory function. *H. erinaceus* has shown promise in managing diabetes, hyperlipidaemia, cancer, and gastric ulcers [1,3]. as well as neurodegenerative diseases such as Alzheimer’s disease [3].

Mushrooms are utilised in the food industry either directly as primary ingredients or additives, or indirectly as fermentation agents [6]. While the use of *H. erinaceus* in food production is still limited, its potential as a source of bioactive compounds and antioxidants has been demonstrated in products such as bread [7], fermented wine and vinegar [8], and non-alcoholic beverages [9]. Additionally, *H. erinaceus* extracts are incorporated into various health products such as meal replacement powders, chewable tablets, and beverages, marketed for gastrointestinal and immune support, with no reported side effects [1].

This study explores the incorporation of Lion’s Mane mushroom as a nutritional additive in oat milk-based desserts tailored for individuals with dysphagia, a condition characterised by difficulty in swallowing solids and liquids [10]. Dysphagia is increasingly recognised as a common condition among older adults, even in those who are otherwise healthy. Recent studies report that around 30% of community-dwelling older adults and up to 50% of residents in aged care facilities experience swallowing difficulties [11]. This condition is closely linked to malnutrition, aspiration pneumonia, and reduced quality of life. Age-related changes in muscle strength and sensory function further contribute to swallowing impairment [12] Soft-textured foods like milk-based desserts are particularly suitable for dysphagic individuals, supporting both nutritional intake and hydration [13].

Texture-modified diets for dysphagia often have low nutrient density, unappealing texture, and poor acceptance, increasing malnutrition risk among older adults [14]. Mushroom powders can enhance gel-based foods by improving water-holding capacity and texture, supporting both nutritional and sensory quality. Ingredient selection and fortification levels should align with nutrient targets and sensory acceptance, with moderate inclusion levels preferred [15]. Despite growing market demand, many commercial products remain high in salt and low in protein and energy [16]. Thus, there is a strong need for nutrient-dense, palatable, and texture-appropriate foods for dysphagia management. The LMP-enriched dessert developed in this study addresses this gap by delivering plant-based protein and improved texture functionality.

This study evaluated the physicochemical characteristics of *H. erinaceus* powders with varying particle sizes, identifying the most suitable for incorporation into oat milk-based desserts. Different concentrations were tested to assess their effects on texture, nutritional quality, and overall product performance. The findings highlight the potential of Lion’s Mane as a sustainable, functional ingredient that meets growing consumer demand for natural and healthy food additives.

## 2. Materials and Methods

Fresh Lion’s Mane mushrooms (*Hericium erinaceus*) were sourced from a XOTIC Mushrooms Farm, Melbourne, Victoria, Australia. Low-fat oat milk was purchased from a local market (“Coles oat milk”, polyunsaturated fat 0.39%, monounsaturated fat 1.47%, saturated fat 0.29%, trans fat 0.09%). Bovine gelatin and iota-carrageenan (IC) were purchased from Melbourne Food Ingredient Depot (Melbourne, VIC, Australia). Ethanol, Folin–Ciocalteu reagent, 2,2-diphenyl-1-picrylhydrazyl, 2,2-azino-bis (3-ethylbenzothiazoline-6-sulphonic acid), Analytical grade Trolox, and gallic acid were purchased from Merck Sigma-Aldrich Pty. Ltd. (Melbourne, VIC, Australia). The total Dietary Fibre kit was purchased from Megazyme (Neogen Australasia, Ipswich, QLD, Australia).

### 2.1. Production of Lion’s Mane Mushroom Powder (LMP)

Fresh mushrooms were dried using a freeze dryer (VaCo 10—Zirbus Technology, Ezzi Vision, Bad Grund, Germany) and then ground into a fine powder using a blade-type coffee grinder for 2 min in 10 s pulses. The resulting mushroom powder was sieved manually using a set of stainless-steel sieves to obtain average particle sizes of 43, 136, and 210 μm as measured by a Mastersizer equipped with a dry cell (3000 ATA, Malvern Panalytical, Malvern, UK) and stored in airtight plastic bags at −18 °C for two weeks prior to further experimentation.

### 2.2. Methods for Analysis of LMP

#### 2.2.1. Chemical Composition, Mineral Content, and Amino Acid Analysis

Moisture, ash, and protein were measured according to the AACC standard [17]. Total dietary fibre was analysed according to the AOAC 991.43 procedure, with modifications, using the Megazyme Total Dietary Fibre (K-TDFR) kit. The amino acid analysis of LMP was tested using standard methods by an NATA-accredited lab (Australian National Measurement Institute, Melbourne, VIC, Australia). The amino acid score (AAS) for essential amino acids was calculated using Equation (1) [18]. The reference essential amino acids were those recommended by FAO/WHO/UNU (2007) [19].

(1) $$AAS\;\left(\%\right)=\frac{Amino\;acid\;per\;100\;g\;test\;protein\;(g)}{Amino\;acid\;per\;100\;g\;reference\;protein\;(g)}\times 100$$

#### 2.2.2. Physical Properties

A chromameter (Chromameter CR—400, Konica Minolta, Tokyo, Japan) was used to measure the colour parameters of the samples, determining the parameters L* (lightness or whiteness, black = 0, white = 100), a* (redness > 0, greenness < 0), b* (yellowness > 0, blue < 0) for each sample according to Umaña et al. (2021) [20]. The bulk density of LMP was measured following the method described by Singh & Liu (2021) [21]. To determine the pH value, 1 g of LMP was added to 9 mL of distilled water and mixed using a magnetic stirrer. The pH was measured at 21 °C using a calibrated pH meter (Seven Compact S220, Mettler Toledo, Switzerland). The water and oil absorption tests were conducted by centrifuging at 5000 rpm for 15 min at 25 °C, following the method described previously [18]. The water solubility test was performed by collecting the supernatant from the water absorption test and oven-drying it at 105 °C for 24 h, as described before [18].

#### 2.2.3. Bioactive Compounds

The Ergocalciferol (Vitamin D2) analysis of LMP was tested using standard methods by an NATA-accredited lab (Australian National Measurement Institute, Melbourne, VIC, Australia) using the VL454 method, which is based on liquid chromatography–tandem mass spectrometry (LC–MS/MS). Total phenolic content (TPC) was determined using the Folin–Ciocalteu method at 765 nm, following a previous method with modifications [3]. Results were expressed as mg gallic acid equivalents per gram of dried sample (mg GAE/g).

The free radical scavenging activity (DPPH assay) and 2,2’-azino-bis (3-ethylbenzothiazoline-6-sulphonic acid (ABTS) assay were measured. Absorbance at 517 nm for the DPPH assay and 743 nm for the ABTS assay was measured using a CLARIOstar^®^ microplate reader (BMG Labtech, Ortenberg, Germany). The results were expressed as mg Trolox equivalents (TE) per gram of dry weight extract (mg TE/g) [3].

#### 2.2.4. Analysis by Fourier Transform Infrared Spectroscopy (FTIR)

FTIR analysis of the LMP was conducted using a spectrometer (GLADIATR, PIKE Technologies, Madison, WI, USA) following the method of Gong et al. (2022) with modifications [22]. Light absorption was measured between a wavenumber range of 4000 to 400 cm^−1^. The spectra were baseline-corrected, and the final spectra represented the average of 64 scans.

### 2.3. Preparation of the Dessert Samples

Dessert samples were prepared based on the method of Majzoobi et al. (2016), with modifications [23]. Table 1 outlines the formulations used. Control samples contained 90% oat milk and 10% sugar, with either 1% or 2% (*w*/*w*) gelatin or iota-carrageenan (IC). Preliminary tests showed that LMP with particle sizes of 136 and 210 μm produced a coarse texture and darker colour, so these were excluded. Aldo, it was found that the addition of more than 5% LMP resulted in an extremely hard, sticky texture and dark colour, making it unsuitable for producing a soft dessert appropriate for dysphagia. The optimal particle size (average 43 μm) was selected, yielding a soft, uniform texture. LMP was added at 2.5% and 5% (*w*/*w*) to one-third of the oat milk and hydrated at 4 °C for 2 h. Concentrations above 5% produced a thick, dark, sticky mixture and were not used. The remaining oat milk was heated to 53 °C, and gelatin or IC and sugar were added with stirring. The mixture was then heated to 65 °C, homogenised (Ultra-Turrax, 15,000 rpm, 5 min), and combined with the hydrated LMP mixture to ensure uniform dispersion of LMP and gelling agents. This shear rate was selected based on preliminary trials, which indicated efficient blending without visible phase separation or texture degradation. The temperature was monitored to ensure it did not exceed 30 °C, with short cooling intervals applied between runs to minimise potential heat buildup and oxidation of bioactive compounds. The mixture was further heated to 72 °C for 30 min to ensure complete gelation and microbial safety without significantly compromising the bioactive integrity of the LMP. Samples were poured into cups, cooled, and stored at 4 °C for 24 h before analysis. Two additional samples (5LMP and 10LMP) were prepared without gelling agents to assess LMP’s gelling ability.

### 2.4. Analysis of the Dessert Samples

#### 2.4.1. Determination of Dry Matter, Colour, and pH of the Dessert Samples

The dry matter content, colour, and pH values of the prepared dessert samples were determined following the method described previously [23].

#### 2.4.2. Measurement of Syneresis

Syneresis of the samples was assessed by using a centrifuge (Sigma 3–30KS centrifuge, Kamakura, Japan) on the 1st, 7th, and 14th day of storage at 4 °C according to Qin et al. (2024) [10]. A 20 g sample was placed in 50 mL centrifuge tubes and centrifuged at 3300× *g* for 15 min at 4 °C.

#### 2.4.3. Texture Profile Analysis (TPA)

The textural properties were determined using a Texture Analyser (TA.XT plus, Stable Micro Systems Ltd., Guildford, UK) equipped with a P/25 probe under a compression mode according to Qin et al. (2024) with some modifications [10]. All tests were performed at a test speed of 0.25 mm/s, with pre- and post-test speeds of 5 mm/s, and a trigger force of 0.049 N. This test speed was selected to allow precise measurement of deformation in the dessert samples, minimising sample rupture during compression. Hardness, springiness, chewiness, cohesiveness, and gumminess were recorded at 25 °C.

#### 2.4.4. Assessing Dysphagia-Appropriateness of Formulated Desserts

The fork pressure and spoon tilt tests were performed to evaluate the desserts for dysphasia and classify them across eight levels (ranging from 0 to 7) according to the International Dysphasia Diet Standardisation Initiative (IDDSI) protocol [24]. To assess their deformation behaviour, the fork pressure test was used, and the refrigerated (4 °C) dessert cubes with a side length of 15 mm were compressed as indicated by thumbnail blanching, in accordance with the IDDSI protocol [24]. The spoon tilt test was used to examine the adhesiveness and cohesiveness of the samples by observing how the scooped samples behaved when the spoon was tilted [24]. Manual IDDSI texture tests were conducted by two assessors. Both assessors independently evaluated the samples and cross-checked the outcomes to ensure consistency and compliance with the IDDSI protocol.

The instrumental fork pressure test was performed using a Texture Analyser (TA.XT plus, Stable Micro Systems Ltd., Guildford, UK) [24]. The numerical data obtained from these tests were aligned with the results from manual IDDSI testing. A fork attachment, developed previously [25]), was fitted to the texture profile analyser and used for the tests. Cube samples with a 15 mm side length were prepared for the analysis. The “hold until time” test sequence of the texture profile analyser was used, applying a constant pressure of 23 N to simulate the manual pressure described in the IDDSI framework, which corresponds to the approximate force exerted during thumbnail blanching. Measurements were taken at a testing speed of 1 mm/s and a pre- and post-test speed of 3 mm/s. The recorded parameters included positive area (Ns^−1^), negative area (Ns^−1^), and positive distance (mm).

### 2.5. Statistical Analysis

Sample data was analysed by One-way analysis of variance (ANOVA) & Tukey test. The mean value and significant differences were compared under a 95% confidence level (*p* < 0.05) by using the software Minitab^®^ 19.2020.1.

## 3. Results and Discussion

### 3.1. Analysis of LMP

#### 3.1.1. Proximate Analysis

The proximate composition of mushrooms can be affected by various factors, including species, developmental stage, variety, sampled part of the mushroom, nitrogen availability, substrate composition, and cultivation practices [26]. LMP with various particle sizes contained a protein content of 15.4–16.6% (Table 2), making it a promising ingredient for the growing demand in vegan/vegetarian food production by offering a sustainable and nutritious alternative to traditional protein sources. The protein content of Lion’s Mane mushrooms previously reported ranged between 7.0–22.3% [22,26,27,28]. The determined protein content in this study was higher compared to other mushroom varieties, such as *Laetiporus sulphurous* (Chicken of the woods) at 8.62%, *Macrolepiota procera* (Parasol mushroom) at 8.56%, and *Polyporus tenuiculus* (Tropical white polypore) at 10.89% [29]. However, the protein content was lower compared to common varieties such as *Agaricus bisporus* (Button mushroom) at 19.13% [20], *Pleurotus ostreatus* (Oyster mushroom) at 19.9–31.2%, *Pleurotus eryngii* (King trumpet) at 22.9–23.2%, and *Pleurotus pulmonarius* (Lung oyster) at 30.5% [30]. In our study, protein content increased slightly (by 1.2%) in samples with smaller particle size, likely due to improved protein extraction and concentration resulting from increased surface area and the breakdown of cellular barriers [31], or possibly due to the uneven distribution of protein and fibre across different parts of the mushroom, which requires further investigation.

LMP was a rich source of fibre (~31%), and the total fibre content was slightly increased (1.78%) with the smaller particle size (Table 2). Previous studies have reported varying fibre contents for Lion’s Mane mushroom, including 3.3–7.8% [32,33], 10.89–11.68% [22], and 57.50% [28]. The variations in the fibre content reported in previous studies may be attributed to differences in cultivation conditions, such as substrate composition, environmental factors, and harvesting stages, all of which can influence the growth and metabolic activity. Furthermore, disparities in analytical methodologies, including sample preparation and extraction techniques, may also contribute to the reported fibre content values. The fibre content observed in this study was higher than that of other mushrooms, such as that of *Cantharellus lutescens* (Yellow Foot) at 15.87% [27] but lower than that of *A. bisporus* at 42.05% [20]. Mushroom fibres offer several advantages over other fibre sources due to their functional bioactivity and inherent antimicrobial and immunomodulatory properties, due to their high β-glucan content [6,34].

The ash content of LMP was ~8% and remained mainly similar for different samples (Table 2). The values obtained in this study were higher than those reported in previous studies, including 6.91% [26], 4.98% [28], and 7.1% [32]. However, the ash content was lower compared to other mushroom species, such as *A. bisporus* (9.59%) [20] and *Craterellus cornucopioides* (Horn of Plenty) (8.37%) [27]. The ash content of mushrooms is an important nutritional indicator and directly reflects the total amount of mineral elements present in the mushroom after incineration.

The moisture content of LMP samples ranged narrowly from 5.33% to 5.48% across particle sizes (Table 2), indicating consistent and well-controlled freeze-drying. The uniformity also suggests that particle size reduction had minimal impact on moisture retention. These low moisture levels, typical of dried mushrooms, support product stability during long-term storage without the need for refrigeration.

Although LMP is generally regarded as safe, rare allergic reactions and potential interactions with medications should be considered, particularly in elderly populations who may be on multiple drugs [35].

#### 3.1.2. Amino Acid Analysis

Amino acid analysis (Table 2) identified eight essential amino acids in LMP, with leucine, lysine, threonine, valine, and phenylalanine being the most abundant. Notable levels of non-essential amino acids, such as glutamic acid, aspartic acid, alanine, arginine, and serine, were also present, supporting the potential of Lion’s Mane as a functional food. Particle size had minimal impact on amino acid composition, though slight variations may be linked to extraction and milling effects, suggesting the need for further optimisation. Amino acid score (Table 3) showed adequate or surplus levels of threonine (149.11%) and phenylalanine + tyrosine (110.11%), but a deficiency in methionine + cysteine (36.36%), consistent with typical limitations of non-animal protein sources.

Threonine is an essential amino acid abundant in LMP (AAS of 149.11%), plays a vital role in immune function, collagen and elastin production, and fat metabolism in the liver [8,36,37,38]. Additionally, LMP is a valuable source of lysine, with an AAS of 99.90%. Lysine is crucial for collagen and elastin production, tissue repair, wound healing, calcium absorption, and the synthesis of hormones and enzymes [8,36,37,38]. Since the human body cannot synthesise lysine, it must be obtained through diet or supplements. The recommended daily intake is approximately 30–64 mg per kg of body weight. Inadequate lysine intake can contribute to protein-energy malnutrition, a condition more commonly observed in developing countries [39]. Enhancing the nutritional quality of non-animal-based foods by increasing protein content and optimising amino acid profiles is crucial. Fortifying vegan and vegetarian diets with LMP offers a promising strategy, as it supplies essential amino acids often lacking in plant-based diets. While LMP provides valuable nutritional benefits, its distinct mushroom flavour and colour may limit its use in certain food products. Future work could explore sensory optimisation strategies, such as flavour masking or blending with lighter ingredients, to improve consumer acceptance.

The AAS of LMP was 87.94%, indicating it meets nearly 88% of the recommended reference values for human nutrition—significantly higher than that of *Agaricus brunnescens*, which has an average AAS of approximately 51.2% [40], *Tricholoma portentosum* (average AAS ~61.8%), and *Tricholoma terreum* (average AAS ~63.3%) [41]. The average AAS value of LMP was low compared to the animal-based proteins, such as eggs (average AAS ~119%) and pork (average AAS ~145%) [42,43]. While LMP cannot fully replace animal proteins, its rich amino acid profile and additional bioactive compounds make it a promising ingredient for enhancing the nutritional quality of plant-based foods.

### 3.2. Physical Properties of LMP

#### 3.2.1. Colour

**Figure 1:**
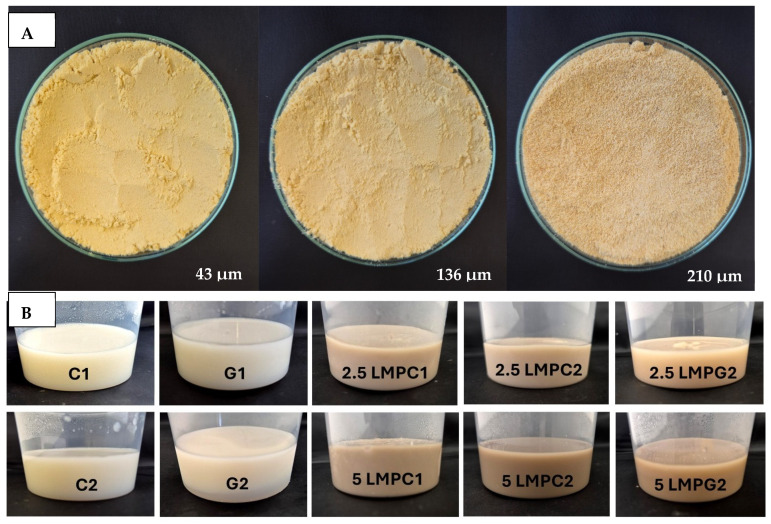
(**A**) LMP samples with different particle sizes; (**B**) Oat milk dessert samples with various formulations. C1: Control with 1% IC, C2: Control with 2% IC, G1: Control with 1% gelatin, G2: Control with 2% gelatin, 2.5LMPC1: 2.5% LMP with 1% IC, 5LMPC1: 5% LMP with 1% IC, 2.5LMPC2: 2.5% LMP with 2% IC, 5LMPC2: 5% LMP with 2% IC, 2.5LMPG2: 2.5% LMP with 2% gelatin, 5LMPG2: 5% LMP with 2% gelatin.

Colour is a key factor in sensory acceptability and consumer appeal, as it influences perceptions of freshness, quality, and nutrition. This is especially important for foods where appearance drives preference. The LMP samples ranged from off-white to light beige (Figure 1A), with the highest lightness (L* = 88.57) in the 43 μm sample (Table 4). The a* values ranged from −1.89 to 0.39, while positive b* values indicated a yellowish hue across all samples. The slightly darker colour in smaller particles may result from drying methods, light exposure, storage, and processing techniques, all of which impact mushroom powder colour [44]. Prolonged milling to obtain smaller particle sizes generates heat, potentially causing browning through the Maillard reaction. Smaller particles can also enhance the extraction of pigments and bioactive compounds due to increased surface area. However, this same surface area makes them more vulnerable to oxidation and degradation, as compounds become more exposed to light and oxygen [45]. In smaller particles, natural pigments like melanin and carotenoids tend to be more concentrated near the surface due to the increased surface area-to-volume ratio caused by particle size reduction. This shift can significantly affect pigment distribution compared to larger particles [46]. This surface enrichment can improve pigment extraction efficiency, leading to a more intense or darker colour compared to larger particles, where pigments are more evenly distributed within the interior matrix [47]. These effects highlight the importance of particle size control in optimising both the appearance and functional properties of mushroom powders.

#### 3.2.2. Bulk Density

Bulk density is a critical parameter reflecting a powder’s filling and packing efficiency, with higher values indicating better compactness [22]. Assessing the bulk density of LMP is essential for evaluating its physical behaviour and its influence on food processing. Density affects flowability, mixing, and distribution within a food matrix, all of which are key to ensuring uniformity and product quality [48]. Consistent density supports the preservation of nutritional and sensory properties while enhancing formulation and manufacturing efficiency. The bulk density of LMP samples increased significantly (*p* < 0.05) with particle size, with the highest value measured for the 210 μm sample as 0.29 g/mL (Table 4). Similar findings were reported previously [22], observing density values of LMP increasing from 0.187 to 0.354 g/mL with the particle size. The lower bulk density of LMP with a smaller particle size may be due to its greater surface area, which leads to looser packing, increased porosity, and more air trapped within the powder. These factors all contribute to a lower overall density compared to larger particle sizes, which tend to pack more tightly and have less void space [49].

#### 3.2.3. pH

The pH of LMP dispersions increased significantly with particle size, reaching 5.48 for the 210 μm sample (Table 4). This mildly acidic pH is primarily due to its production of organic acids such as oxalic and malic acids and its preference for slightly acidic growth environments [50]. Due to its mildly acidic nature, LMP can lower the pH of food products at higher concentrations, potentially impacting their functional properties. A reduced pH may disrupt gelation by affecting electrostatic interactions between carrageenan and milk proteins, and can also lead to changes in texture, increased syneresis, and altered colour or sensory attributes in products such as dairy desserts [51]. Silva et al. (2025) reported a similar pH value for Lion’s Mane mushroom as 5.80 [52]. The pH value can vary with particle size due to increased surface area and the release of soluble compounds. However, this variation is also influenced by factors such as mushroom species, processing methods, and the internal distribution of pH-affecting compounds within the mushroom matrix [31,53].

#### 3.2.4. Water Solubility, Water Absorption, and Oil Absorption Capacity

The water solubility of LMP samples decreased significantly (*p* < 0.05) with increasing particle size. The highest solubility value was measured for the 43 μm sample as 34.64% (Table 5). It has been reported that the water solubility of Lion’s Mane mushroom increased with decreasing particle size, ranging from 41.67% to 53.33% [22]. This increase is likely due to the greater surface area achieved through grinding, which enhances particle–water interactions. Additionally, grinding may cause structural modifications, converting some insoluble components into soluble forms, thereby improving overall solubility [54]. Umaña et al. (2021) reported the water solubility of *A. bisporus* as 44.93%, indicating that LMP exhibits slightly lower solubility than some other mushroom species [20].

LMP showed a remarkable water absorption capacity (243.67% to 340.01%), indicating the capacity of LMP as a water absorber and a potential thickening agent. The water absorption capacity increased significantly (*p* < 0.05) with reduced particle sizes. The high water uptake of the LMP can be attributed to the abundant proteins, fibres, and other hydrocolloids that can absorb water [53,54]. It may also be attributed to filling the voids of the porous structures formed during freeze drying. The samples also showed high oil absorption capacity, suggesting that LMP can be used as a valuable ingredient for improving the texture and mouthfeel of oil-containing food products. The values decreased significantly (*p* < 0.05) with increasing particle size. The highest oil absorption value was measured for the 43 μm sample as 250.89% (Table 5).

The increased water solubility, water absorption, and oil absorption capacity of LMP with decreasing particle size can be attributed to the larger surface area, improved wettability, enhanced capillary action, and greater exposure of functional groups such as proteins, fibre, and hydrocolloids in the smaller particles. Similar results were obtained in a study conductedon *Hypsizygus marmoreus* mushroom powder [31].

### 3.3. Bioactive Compounds

#### 3.3.1. Vitamin D_2_ (Ergocalciferol) Content

Mushrooms are a rich source of ergosterol, a key structural component of fungal cell membranes and the precursor to vitamin DVitamin D is vital for calcium metabolism and bone health, existing in two main forms: cholecalciferol (D_3_) from animal sources and ergocalciferol (D_2_), which is synthesised in fungi through UV irradiation of ergosterol. In Lion’s Mane mushroom, reported ergosterol levels range from 2.00 to 2.52 mg/g [5,55]. As plant-based sources of vitamin D are scarce, vegetarians are particularly susceptible to deficiency unless supplemented. Mushrooms offer a natural, dietary source of vitamin D_2_ suitable for vegan/vegetarian diets to meet the recommended daily intake of 15 µg/day [30,32]. In this study, LMP contained almost the same vitamin D_2_ content across different particle sizes (0.5–0.6 µg/100 g). Previous studies have reported that other mushrooms, such as shiitake and oyster, generally contain less than 1 µg/100 g of vitamin D_2_ [56]. The low vitamin D_2_ content observed in LMP is consistent with mushrooms that have not been exposed to ultraviolet (UV) light during cultivation or processing. Vitamin D_2_ is synthesised from ergosterol in mushrooms upon exposure to UV-B or UV-C light; without such exposure, only trace amounts are naturally present [32].

#### 3.3.2. TPC

The content of phenolic compounds in mushrooms is influenced by several factors, such as extraction methods, growth conditions (moisture levels, light exposure), harvest maturity stage, and species variability. Among the most commonly identified phenolic acids in LM extracts are gallic acid, *p*-coumaric acid, and *p*-hydroxybenzoic acid [3]. These polyphenols contribute to the potential therapeutic and functional properties of LMP, making it a promising ingredient for health-promoting food formulations.

In this study, the TPC of LMP showed a significant decrease (*p* < 0.05) with increasing particle size, ranging from 72.15 to 58.01 mg GAE/g (Table 5). This reduction in TPC with larger particle sizes may be attributed to the decreased surface area available for extraction, as finer particles provided greater exposure to solvents, enhancing polyphenol extraction and detection. TPC values have been reported in previous studies for Lion’s Mane, ranging from 7.42–13.5 µg GAE/mg extract [2], 2.17 mg GAE/g [57], 17.10 mg GAE/g [58], and 753 mg catechol eq./g [26]. Additionally, studies on other mushroom species, such as *Ganoderma lucidum* and *Agrocybe aegerita*, have reported total phenolic content values of 28.11 mg GAE/g and 16.05 mg GAE/g, respectively [58]. The TPC of LMP was up to 10 times higher than that of other samples, indicating a significantly greater concentration of phenolic acids, compounds known for their strong antioxidant and free radical scavenging properties. This elevated phenolic content in Lion’s Mane has been linked to enhanced antioxidant activity and potential neuroprotective effects [3], supporting its role in reducing oxidative stress and contributing to overall health benefits.

#### 3.3.3. Antioxidant Assays

In this study, DPPH values exhibited a slight decrease as particle size increased, ranging from 42.27 to 38.39 mg TE/g (Table 5). Similar findings were reported previously [22], with improved antioxidant activity likely due to greater surface area exposure and improved extraction efficiency of smaller particle sizes. DPPH values have been reported in previous studies for Lion’s Mane, with findings such as 985–1900 EC_50_ μg/mL [2], 13.93% [57], and 2.30 IC_50_ mg/mL [59]. Lower DPPH values have been reported for other mushroom species, including *A. bisporus* (0.13–1.53 μM TE/g), *P. ostreatus* (0.08–1.52 μM TE/g), and *Inonotus hispidus* (1.72–9.50 μM TE/g) [60].

The ABTS assay is another widely used method for evaluating the free radical scavenging capacity of plant extracts, particularly for both hydrophilic and lipophilic antioxidant compounds. This assay functions by measuring the ability of polyphenols to donate hydrogen ions, neutralising ABTS radicals in the process. One key factor influencing ABTS scavenging activity is the drying method, as prolonged exposure to low temperatures during freeze-drying can lead to partial degradation of antioxidants, potentially reducing their effectiveness. In this study, the ABTS values of LMP ranged from 23.30 to 24.16 mg TE/g (Table 5). ABTS values have been reported in previous studies for Lion’s Mane, with findings such as 300–1200 EC_50_ μg/mL [2] and 1.72 IC_50_ mg/mL [59]. Lion’s Mane mushroom demonstrated superior antioxidant capacity compared to several other commonly studied species. For instance, reported ABTS values for *Agaricus bisporus* (0.82–2.06 μM TE/g), *Pleurotus ostreatus* (0.10–0.80 μM TE/g), and *Inonotus hispidus* (5.87–165.00 μM TE/g) are generally lower or more variable [60]. This suggests that Lion’s Mane may offer superior antioxidant potential, supporting its use as a functional ingredient in health-promoting food applications. Variations in antioxidant activity and content reported in previous studies reflect the influence of factors such as mushroom species, extraction methods, and particle size on the antioxidant capacity of mushroom powders.

Due to time constraints, the antioxidant potential of the final dessert was not determined in this study. The heating and homogenization steps used in dessert preparation may partially reduce the activity of heat-sensitive antioxidants, and interactions with other matrix components could further influence their release and bioavailability. Future studies should systematically evaluate antioxidant retention and bioavailability in LMP-enriched desserts, considering both processing conditions and matrix interactions, to optimise the functional potential of these texture-modified products.

### 3.4. FTIR

**Figure 2:**
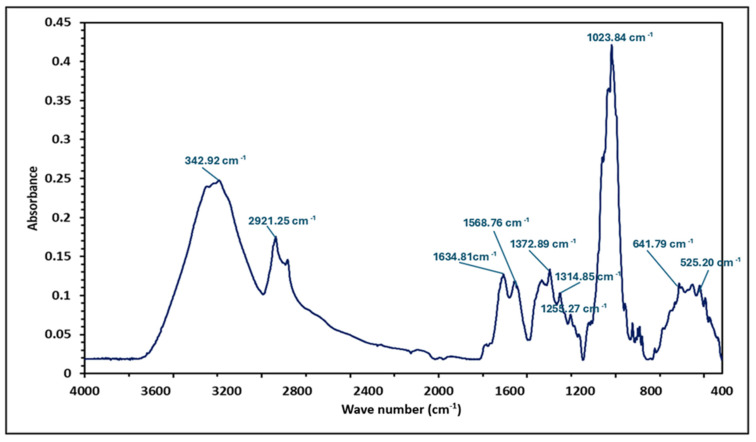
FTIR spectra of LMP (43 μm particle size) illustrating the absorbance at different wavenumbers.

FTIR was used to identify and analyse the chemical composition and functional groups of the LMP (Figure 2). The 43 µm particle size was selected due to its finer texture, which ensured better sample homogeneity and minimised light scattering, leading to more reliable and reproducible results. The FTIR analysis revealed a wide range of absorbance peaks between 400 and 4000 cm^−1^, confirming the presence of diverse chemical compositions in LMP, including proteins, lipids, and carbohydrates. The broad absorption peak around 3242 cm^−1^ corresponded to the stretching vibration of O-H bonds, which are commonly found in fibre and polyphenolic structures [22]. The peak at 2921 cm^−1^ was attributed to CH_2_ and CH_3_ stretching vibrations, likely originating from fatty acids present in the cell wall [26]. The bands between 1640–1580 cm^−1^ were linked to the bending vibrations of H_2_O molecules [22]. Two major bands at 1634 cm^−1^ and 1568 cm^−1^ corresponded to amide I and amide II bands, respectively, which are characteristic of protein structures [26]. The region between 1200 and 1000 cm^−1^, including a sharp peak at 1023 cm^−1^, represented stretching vibrations of C–O and C–O–C bonds in polysaccharides. The 950–750 cm^−1^ range was associated with the anomeric configuration of polysaccharides [22,26]. The 750–1200 cm^−1^ region served as a fingerprint region to distinguish different mushroom species, while the 1200–1000 cm^−1^ spectral region was particularly useful for identifying the mushroom genus [61].

### 3.5. Visual Properties of the Dessert Samples

Oat milk was chosen as the base for the dessert due to its popularity as a plant-based dairy alternative in vegan and health-conscious diets. Its stable emulsion, mild flavour, and smooth, creamy texture make it well-suited for a variety of applications, including desserts [62]. IC was selected in this study as a substitute for gelatin in certain samples, targeting use in vegan diets. IC is known for forming soft, elastic gels in the presence of calcium ions and is commonly used in plant-based products and dairy alternatives [63]. To develop a soft gel suitable for dysphagia-friendly desserts, preliminary trials were carried out with three LMP particle sizes. The 43 μm sample produced a smooth texture, while larger particles resulted in coarse, grainy textures and were excluded. Samples with 5% and 10% LMP were thick but unable to retain shape, indicating the need for additional gelling agents such as gelatin or iota carrageenan. Therefore, these two samples were not considered for further testing, as they were not set properly. LMP concentrations above 5% produced sticky, dark mixtures with poor sensory appeal, aligning with previous findings on observed texture deterioration in products containing more than 5% mushroom [32]. As a result, oat milk desserts were formulated with 43 μm LMP at 2.5% and 5% to achieve a soft, stable gel suitable for dysphagia.

### 3.6. Analysis of the Prepared Dessert Samples

#### 3.6.1. Dry Matter

Determining dry matter is important for assessing the texture, shelf-life, and nutritional value of desserts. The dry matter percentage significantly increased (*p* < 0.05) with the incorporation of LMP, IC, and gelatin, indicating their contribution to the total solid content in the dessert formulation (Table 6). The highest dry matter value was measured as 27.29% for the sample 5LMPCMajzoobi et al. (2016) observed a rise in dry matter from 21.57% to 29.43% when increasing wheat germ levels in dessert formulations [23]. The increase in dry matter can be attributed to the binding and gelling properties of the added ingredients, which contribute to the structural integrity and texture of the final product.

#### 3.6.2. pH

With the incorporation of LMP, the pH values of dessert samples decreased significantly (*p* < 0.05), indicating a shift towards higher acidity. The lowest pH value, measured as 5.67, was observed in sample 5LMPC1 (Table 6). This reduction in pH can be attributed to the high content of fatty acids and amino acids in LMP, particularly glutamic acid and aspartic acid, which are naturally acidic. The presence of antioxidants with acidic properties further contributes to this decrease, influencing the overall acidity, stability, and sensory properties of the dessert formulations [23].

#### 3.6.3. Colour

The colour values of the developed dessert samples varied depending on the levels of LMP, IC, and gelatin (Figure 1B). The lightness decreased significantly (*p* < 0.05) with the incorporation of LMP, likely due to its natural pigmentation (Table 6). IC incorporated control samples exhibited higher lightness values compared to gelatin incorporated control samples, suggesting that IC contributed to a brighter appearance. The a* and b* values of LMP incorporated samples increased, indicating a shift towards red and yellow hues compared to the control samples.

#### 3.6.4. Syneresis

**Figure 3:**
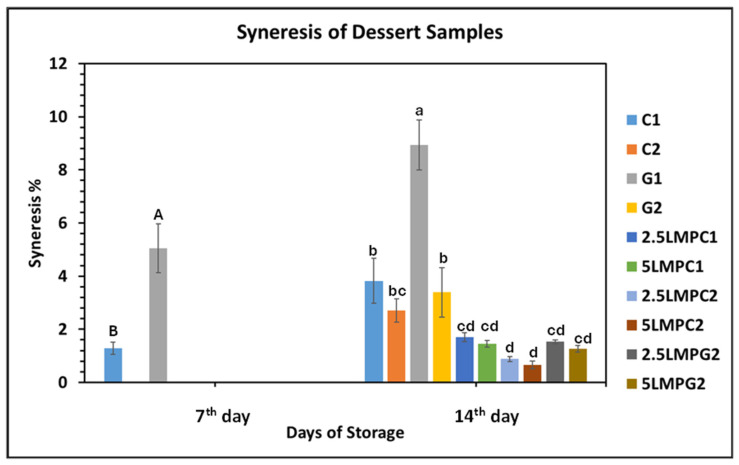
Syneresis of the prepared dessert samples. Means with a different letter in the same column are significantly different (*p* < 0.05).

Syneresis measurement in desserts is a key indicator of their ability to prevent unwanted liquid separation during refrigeration. It occurs when water is expelled from the gel network and is influenced by hydrocolloids and molecular interactions within the gel matrix. Water-binding components like hydrocolloids and sugars reduce syneresis, improving dessert stability and mouthfeel. In gel systems, specific polysaccharides interact with proteins to enhance hydration and gel stability. Excessive syneresis compromises gel integrity and shortens product shelf-life [10,64]. The dessert samples showed no syneresis on day 1 but developed varying levels by days 7 and 14 (Figure 3). On day 7, only the G1 and C1 samples exhibited low syneresis at 5.05% and 1.29%, respectively. By day 14, the highest syneresis occurred in the G1 sample (8.94%), indicating lower stability. Samples containing LMP had significantly less syneresis (*p* < 0.05) than controls, likely due to the water-binding effects of LMP’s proteins and fibre, which improved water retention and structural integrity. Additionally, the samples with iota carrageenan showed lower syneresis than those with gelatin. This aligns with previous findings on IC-incorporated milk puddings [64]. The superior water retention of IC is likely due to its stronger gel-forming ability and higher water-binding capacity, which help maintain the structural stability, reducing liquid expulsion during storage.

#### 3.6.5. Texture Profile Analysis

Texture is crucial for food preference and acceptance, especially among the elderly. Texture profile analysis (Table 7) showed that increasing levels of LMP, IC, and gelatin significantly raised hardness (*p* < 0.05). However, gelatin desserts with LMP (2.5LMPG2 and 5LMPG2) had lower hardness than the gelatin-only control (G2). The highest hardness (132.08 g) was in the 5LMPC2 sample. Hardness affects mouthfeel and correlates with gumminess—the energy needed to break down a semi-solid. LMP addition to IC-based desserts strengthened the gel network, increasing mechanical strength and the energy required for swallowing, making these desserts suitable for individuals with dysphagia [10]. In addition, LMP contains a high amount of protein and fibre, which can bind a large amount of water due to the presence of hydroxyl groups. This leads to reduced free water content and increased stiffness in the desserts [23].

Springiness in dessert samples refers to their ability to regain their original shape after compression, reflecting the product’s elasticity or resilience, a key textural attribute. In this study, springiness values did not show any significant differences among the samples. As the levels of IC and gelatin increased, cohesiveness values decreased significantly (*p* < 0.05). Samples with 2.5% LMP exhibited higher cohesiveness compared to those with 5% LMP. The highest cohesiveness value (0.85) was observed in sample 2.5LMPCCohesiveness reflects how easily a food bolus deforms during swallowing, influencing swallowing ease. Adequate cohesiveness is crucial to prevent food residue in the pharynx and ensure safe swallowing. Carrageenan helps form internal bonds that hold the bolus together, reducing breakdown into small particles and minimising choking risk [10].

Gumminess refers to the energy needed to break down a semi-solid food into a swallowable form, while chewiness measures the energy required during chewing before swallowing. Since elderly individuals with swallowing difficulties often have limited oral strength, foods with lower chewiness can pass more easily through the pharynx without stickiness, improving both safety and sensory appeal [10]. Gumminess and chewiness showed similar trends, increasing significantly (*p* < 0.05) with higher IC and gelatin levels. However, in IC-containing samples, both values decreased significantly (*p* < 0.05) as LMP concentration increased. This suggests that interactions between LMP and other ingredients may affect texture, warranting further study. Similar results have been reported with the addition of wheat germ to fresh chilled dairy desserts by [23].

#### 3.6.6. Dysphasia Appropriateness Test

The International Dysphagia Diet Standardisation Initiative (IDDSI) Framework (2019) [65] offers a classification system for food textures, detailing consistency levels from thin to extremely thick, specifically designed to support individuals with dysphagia [24]. The framework includes qualitative tests like the fork pressure and spoon tilt methods to assess food and liquid textures. However, since these rely on visual and subjective assessments without standardised instruments, they do not provide numerical data and may yield inconsistent or inaccurate results. Therefore, using objective, standardised instrumental methods is recommended for greater reliability [10]. Pematilleke et al. (2022) introduced an instrumental method that replicates the IDDSI fork pressure test by using a texture analyser equipped with a fork attachment [25]. This approach offers a reliable alternative capable of producing quantitative measurements. In this study, both the manual (standard) and instrumental IDDSI fork pressure tests, along with the manual spoon tilt test, were applied to the prepared samples. The results, including numerical data and photographic observations, are presented in Table 8 and Table 9.

During the manual fork pressure test (Table 8), all samples deformed easily under pressure and did not regain their original shape after fork removal. Accordingly, all custard samples were classified as Level 6—Soft & Bite-Sized per IDDSI guidelines and considered safe with no choking risk [65]. Samples with higher IC and gelatin levels required more force to compress and showed firmer textures with deeper fork marks, reflecting the enhanced gel network entanglement provided by these hydrocolloids.

The instrumental fork pressure test was conducted on all samples, and the results are shown in Table Three key parameters (peak positive distance, positive area, and negative area) were measured using a texture profile analyser. The peak positive distance refers to how far the fork moved or the point of maximum force on the force-time curve, which is influenced by the hardness of the food [25]. This distance increased with higher levels of IC, gelatin, and LMP, aligning with the findings from the visual observations in the IDDSI test (Table 8) and texture results (Table 7). The positive area, which reflects the time taken to reach the target force [25], also rose with increasing IC, gelatin, and LMP contents, indicating higher hardness in the samples (Table 9). The negative area represents the adhesiveness of the sample as it separates from the fork. Sample G1 recorded the highest negative area, suggesting high stickiness. In contrast, samples with elevated IC and gelatin levels had lower negative area values, indicating reduced adhesiveness and suggesting they are less sticky and easier to swallow (Table 9). These findings show that the instrumental fork pressure test eliminates human subjectivity, enhances measurement precision, and yields dependable data for assessing the level 6 dysphagia-specific foods [10].

The spoon tilt test was performed on all dessert samples to evaluate stickiness and their ability to hold shape. This test involves tilting a spoonful of the sample to see if it stays in place or slides off [64]. All samples maintained their form and left no residue on the spoon after tilting (Table 8), a favourable characteristic for dysphagia-friendly foods as it helps ensure easy swallowing without sticking to the tongue or throat [24]. Based on IDDSI guidelines, all samples were classified under Level 6—Soft & Bite-sized dysphagia.

## 4. Conclusions

This study highlights the potential of *Hericium erinaceus* as a novel functional ingredient in soft food formulations, particularly for individuals with dysphagia and age-related conditions such as Alzheimer’s disease. Rich in high-quality protein (15.4–16.6% and AAS ~88), fibre (30.10–31.88%), and bioactive compounds, Lion’s Mane mushroom offers both nutritional and neuroprotective benefits. A practical serving size of 100 g of the 5% LMP dessert would provide approximately 0.8 g of additional protein and 1.6 g of dietary fibre. Incorporating up to 5% freeze-dried LMP into oat milk-based desserts significantly enhanced their nutritional profile and textural suitability for dysphagic diets, without compromising sensory quality. At higher concentrations, however, adverse effects on texture and colour were observed. Iota-carrageenan had a stronger capability to modify texture as compared to gelatin when the same concentrations were employed. The development of carrageenan-based vegan formulations further broadens the applicability of these products. This innovative approach supports the growing demand for plant-based, health-promoting foods and provides a promising foundation for future research into functional soft foods. Continued investigation into optimal incorporation methods, sensory acceptability, and clinical validation will be essential to fully realise the therapeutic and commercial potential of Lion’s Mane in food systems.

## Figures and Tables

**Table 1:** Formulations of the prepared dessert samples.

Sample Name	Gelling Agent (% *w*/*w*)	LMP (% *w*/*w*)
C1	IC * 1%	-
C2	IC 2%	-
G1	Gelatin 1%	-
G2	Gelatin 2%	-
2.5LMPC1	IC 1%	2.5
5LMPC1	IC 1%	5
2.5LMPC2	IC 2%	2.5
5LMPC2	IC 2%	5
2.5LMPG2	Gelatin 2%	2.5
5LMPG2	Gelatin 2%	5
5LMP	-	5
10LMP	-	10

* Iota carrageenan (IC); C1: Control with 1% IC, C2: Control with 2% IC, G1: Control with 1% gelatin, G2: Control with 2% gelatin, 2.5LMPC1: 2.5% LMP with 1% IC, 5LMPC1: 5% LMP with 1% IC, 2.5LMPC2: 2.5% LMP with 2% IC, 5LMPC2: 5% LMP with 2% IC, 2.5LMPG2: 2.5% LMP with 2% gelatin, 5LMPG2: 5% LMP with 2% gelatin, 5LMP: 5% LMP, 10LMP: 10% LMP. “-” have not been added in the formulation.

**Table 2:** Chemical composition and amino acid composition of proteins of LMP with different particle size ranges *.

Particle Size	43 μm	136 μm	210 μm
Ash (%)	8.02	7.96	7.95
Moisture (%)	5.33	5.39	5.48
Protein (%)	15.4	16.3	16.6
Total fibre (%)	30.10	31.53	31.88
Ergocalciferol (vitamin D_2_) (μg/100 g LMP)	<0.5	0.6	<0.5
Essential amino acids (mg/100 g LMP)			
Histidine	180	160	170
Isoleucine	380	380	380
Leucine	810	810	810
Lysine	800	820	710
Methionine	140	150	150
Phenylalanine	400	390	430
Threonine	620	600	620
Valine	530	530	510
Non-essential amino acids (mg/100 g LMP)			
Alanine	890	910	850
Arginine	670	670	690
Aspartic Acid	1200	1200	1100
Glutamic Acid	2200	2100	2000
Glycine	500	500	520
Hydroxyproline	29	30	31
Proline	500	510	530
Serine	650	640	670
Taurine	<5	<5	<5
Tyrosine	380	370	380

* Values are the average of duplicates. The coefficient of variance (CV) of the testing methods was 5%.

**Table 3:** Amino acid scores (AAS) of the essential amino acids in LMP with average particle size of 43 μm, as compared to the recommended reference amino acids.

Essential Amino Acids	Content (mg/g Protein)	Reference Amino Acids (mg/g Protein) ^1^	AAS (%)
Histidine	11.69	18	64.94
Isoleucine	24.68	31	79.60
Leucine	52.60	63	83.49
Lysine	51.95	52	99.90
Methionine + Cysteine	9.09	25	36.36
Phenylalanine + Tyrosine	50.65	46	110.11
Threonine	40.26	27	149.11
Valine	34.42	43	80.04
Average			87.94

^1^ Reference amino acids recommended by FAO/WHO/UNU (2007) reported by Millward (2012).

**Table 4:** pH, bulk density, and colour parameters of LMP.

LMP Particle Size	Colour			Bulk Density (g/mL)	pH
	L*	a*	b*		
43 μm	88.57 ± 0.61 ^a^	−1.89 ± 0.07 ^b^	31.46 ± 0.25 ^b^	0.13 ± 0.01 ^c^	5.33 ± 0.01 ^c^
136 μm	86.48 ± 1.25 ^b^	−2.40 ± 0.19 ^c^	32.16 ± 0.62 ^b^	0.16 ± 0.01 ^b^	5.39 ± 0.01 ^b^
210 μm	82.14 ± 1.17 ^c^	0.39 ± 0.20 ^a^	34.15 ± 0.41 ^a^	0.29 ± 0.01 ^a^	5.48 ± 0.01 ^a^

All analyses were performed in triplicate (*n* = 3). Values are represented as mean ± standard deviation. Different letters within the same column indicate significant differences (*p* < 0.05).

**Table 5:** Water solubility, water absorption, oil absorption capacity and bioactive compounds of LMP.

Particle Size	Water Solubility %	Water Absorption %	Oil Absorption %	TPC (mg GAE/g)	DPPH (mg TE/g)	ABTS (mg TE/g)
43 μm	34.64 ± 0.82 ^a^	340.01 ± 3.06 ^a^	250.89 ± 5.29 ^a^	72.15 ± 3.57 ^a^	42.27 ± 1.58 ^a^	23.89 ± 0.58 ^a^
136 μm	32.52 ± 0.67 ^b^	275.30 ± 5.81 ^b^	218.59 ± 4.13 ^b^	61.71 ± 2.69 ^b^	41.54 ± 0.36 ^a^	24.16 ± 0.19 ^a^
210 μm	22.24 ± 0.38 ^c^	243.67 ± 4.53 ^c^	182.63 ± 7.44 ^c^	58.01 ± 3.43 ^b^	38.39 ± 1.11 ^b^	23.30 ± 1.54 ^a^

All analyses were performed in triplicate (*n* = 3). Values were represented as mean ± standard deviation. Different letters within the same column indicate significant differences (*p* < 0.05).

**Table 6:** Physical properties of the oat milk dessert samples.

Sample	Dry Matter (%)	pH	Colour		
			L*	a*	b*
C1	20.09 ± 0.96 ^g^	7.67 ± 0.02 ^b^	83.04 ± 0.96 ^a^	−1.58 ± 0.13 ^e^	9.96 ± 0.39 ^d^
C2	22.62 ± 0.17 ^de^	7.94 ± 0.09 ^a^	82.90 ± 0.58 ^a^	−1.62 ± 0.26 ^e^	9.42 ± 0.57 ^d^
G1	21.07 ± 0.03 ^fg^	7.23 ± 0.01 ^c^	78.66 ± 0.81 ^b^	−3.10 ± 0.03 ^g^	1.24 ± 0.14 ^f^
G2	21.73 ± 0.11 ^ef^	7.07 ± 0.01 ^d^	77.56 ± 0.44 ^b^	−2.34 ± 0.08 ^f^	7.89 ± 0.35 ^e^
2.5LMPC1	23.38 ± 0.30 ^cd^	6.16 ± 0.03 ^f^	54.26 ± 1.66 ^f^	0.62 ± 0.01 ^c^	13.29 ± 0.39 ^b^
5LMPC1	25.08 ± 0.01 ^b^	5.67 ± 0.08 ^h^	42.75 ± 1.45 ^g^	1.50 ± 0.15 ^b^	11.67 ± 0.94 ^c^
2.5LMPC2	25.28 ± 0.20 ^b^	6.57 ± 0.03 ^e^	67.86 ± 1.95 ^d^	1.46 ± 0.05 ^b^	16.82 ± 0.58 ^a^
5LMPC2	27.29 ± 0.12 ^a^	6.19 ± 0.03 ^f^	60.04 ± 0.46 ^e^	2.37 ± 0.06 ^a^	16.34 ± 0.39 ^a^
2.5LMPG2	23.89 ± 0.19 ^c^	5.81 ± 0.05 ^g^	70.50 ± 1.07 ^c^	0.01 ± 0.04 ^d^	15.80 ± 0.39 ^a^
5LMPG2	25.49 ± 0.59 ^b^	5.72 ± 0.02 ^gh^	34.96 ± 1.18 ^h^	1.30 ± 0.07 ^b^	10.42 ± 0.48 ^d^

All analyses were performed in triplicate (*n* = 3). Values are represented as mean ± standard deviation. Different letters within the same column indicate significant differences (*p* < 0.05). * Iota carrageenan (IC); C1: Control with 1% IC, C2: Control with 2% IC, G1: Control with 1% gelatin, G2: Control with 2% gelatin, 2.5LMPC1: 2.5% LMP with 1% IC, 5LMPC1: 5% LMP with 1% IC, 2.5LMPC2: 2.5% LMP with 2% IC, 5LMPC2: 5% LMP with 2% IC, 2.5LMPG2: 2.5% LMP with 2% gelatin, 5LMPG2: 5% LMP with 2% gelatin.

**Table 7:** Texture profile analysis of the prepared dessert samples.

Sample	Hardness (g)	Springiness (%)	Cohesiveness	Gumminess (g)	Chewiness (N)
C1	28.55 ± 0.18 ^h^	99.98 ± 0.29 ^a^	0.61 ± 0.03 ^c^	17.50 ± 0.80 ^g^	17.53 ± 0.83 ^h^
C2	99.54 ± 5.08 ^c^	99.89 ± 0.22 ^a^	0.45 ± 0.02 ^de^	44.20 ± 1.82 ^d^	44.29 ± 1.85 ^d^
G1	22.97 ± 0.89 ^h^	100.14 ± 0.35 ^a^	0.58 ± 0.01 ^c^	13.37 ± 0.53 ^g^	13.39 ± 0.52 ^h^
G2	104.75 ± 4.00 ^c^	100.15 ± 0.16 ^a^	0.49 ± 0.01 ^d^	51.10 ± 1.65 ^c^	51.18 ± 1.63 ^c^
2.5LMPC1	46.22 ± 4.19 ^g^	100.40 ± 0.04 ^a^	0.85 ± 0.03 ^a^	39.07 ± 3.52 ^de^	39.23 ± 3.52 ^de^
5LMPC1	57.09 ± 3.25 ^f^	100.16 ± 0.51 ^a^	0.46 ± 0.04 ^de^	26.07 ± 1.17 ^f^	26.11 ± 1.11 ^g^
2.5LMPC2	117.78 ± 6.17 ^b^	100.25 ± 0.24 ^a^	0.82 ± 0.03 ^a^	97.04 ± 5.67 ^a^	97.28 ± 5.62 ^a^
5LMPC2	132.08 ± 5.03 ^a^	100.13 ± 0.33 ^a^	0.68 ± 0.05 ^b^	89.78 ± 6.21 ^b^	89.89 ± 6.16 ^b^
2.5LMPG2	70.12 ± 2.86 ^e^	99.92 ± 0.49 ^a^	0.45 ± 0.03 ^de^	31.59 ± 2.82 ^f^	31.55 ± 2.74 ^fg^
5LMPG2	78.82 ± 1.66 ^d^	100.29 ± 0.49 ^a^	0.42 ± 0.02 ^e^	32.80 ± 1.73 ^ef^	32.89 ± 1.80 ^ef^

All analyses were performed in triplicate (*n* = 3). Values are represented as mean ± standard deviation. Different letters within the same column indicate significant differences (*p* < 0.05). Iota carrageenan (IC); C1: Control with 1% IC, C2: Control with 2% IC, G1: Control with 1% gelatin, G2: Control with 2% gelatin, 2.5LMPC1: 2.5% LMP with 1% IC, 5LMPC1: 5% LMP with 1% IC, 2.5LMPC2: 2.5% LMP with 2% IC, 5LMPC2: 5% LMP with 2% IC, 2.5LMPG2: 2.5% LMP with 2% gelatin, 5LMPG2: 5% LMP with 2% gelatin.

**Table 8:** IDDSI test of the prepared dessert samples.

Sample	Fork Pressure Test	Spoon Tilt Test
C1	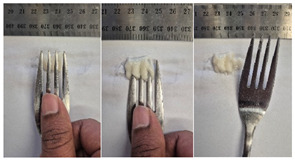	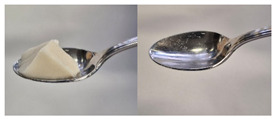
C2	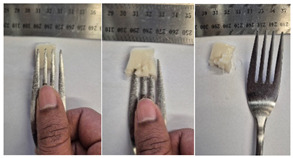	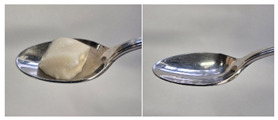
G1	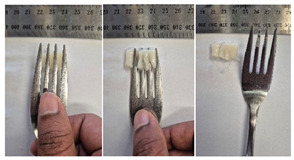	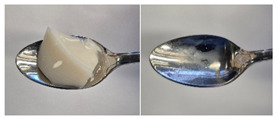
G2	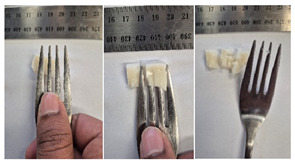	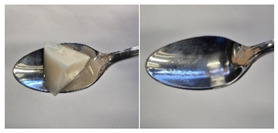
2.5LMPC1	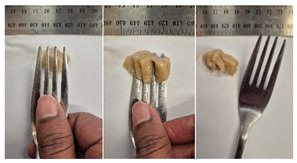	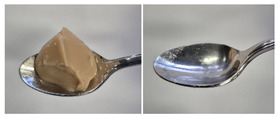
5LMPC1	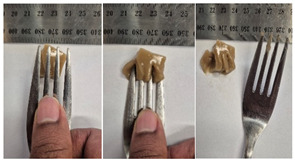	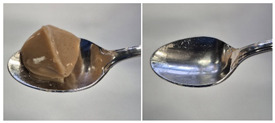
2.5LMPC2	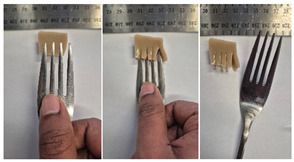	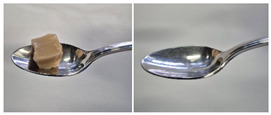
5LMPC2	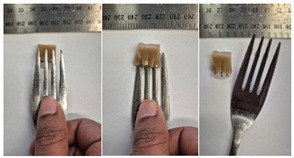	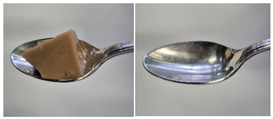
2.5LMPG2	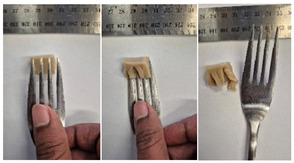	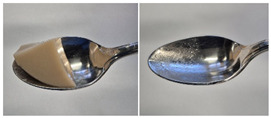
5LMPG2	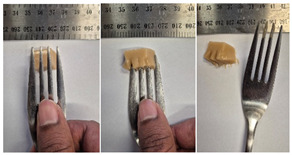	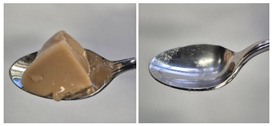

**Table 9:** Instrumental IDDSI test of the prepared dessert samples.

Sample	Peak Positive Distance (mm)	Positive Area (g.s)	Negative Area (g.s)
C1	5.17 ± 0.55 ^c^	12585.00 ± 6.10 ^f^	−37.13 ± 9.15 ^de^
C2	7.94 ± 0.33 ^ab^	12914.70 ± 64.50 ^cd^	−12.31 ± 3.83 ^ab^
G1	5.98 ± 0.63 ^bc^	12578.70 ± 26.80 ^f^	−50.62 ± 3.60 ^e^
G2	8.70 ± 0.81 ^a^	12826.00 ± 121.60 ^cde^	−6.91 ± 2.76 ^a^
2.5LMPC1	6.40 ± 0.34 ^bc^	12636.70 ± 10.40 ^ef^	−29.81 ± 2.45 ^cd^
5LMPC1	8.96 ± 1.50 ^a^	12767.30 ± 93.80 ^def^	−35.04 ± 4.65 ^cd^
2.5LMPC2	9.46 ± 0.39 ^a^	13188.70 ± 146.70 ^b^	−24.07 ± 5.31 ^bcd^
5LMPC2	10.04 ± 0.89 ^a^	13569.70 ± 36.10 ^a^	−28.29 ± 1.58 ^cd^
2.5LMPG2	8.93 ± 0.60 ^a^	13034.70 ± 74.30 ^bc^	−20.42 ± 6.36 ^abc^
5LMPG2	9.68 ± 0.96 ^a^	13438.70 ± 58.40 ^a^	−24.68 ± 6.36 ^bcd^

All analyses were performed in triplicate (*n* = 3). Values are represented as mean ± standard deviation. Different letters within the same column indicate significant differences (*p* < 0.05). Iota carrageenan (IC); C1: Control with 1% IC, C2: Control with 2% IC, G1: Control with 1% gelatin, G2: Control with 2% gelatin, 2.5LMPC1: 2.5% LMP with 1% IC, 5LMPC1: 5% LMP with 1% IC, 2.5LMPC2: 2.5% LMP with 2% IC, 5LMPC2: 5% LMP with 2% IC, 2.5LMPG2: 2.5% LMP with 2% gelatin, 5LMPG2: 5% LMP with 2% gelatin.

## Data Availability

The research data will be available upon request.
